# How we treat octogenarians with brain metastases

**DOI:** 10.3389/fonc.2023.1213122

**Published:** 2023-08-08

**Authors:** Carsten Nieder, Nicolaus H. Andratschke, Anca L. Grosu

**Affiliations:** ^1^ Department of Oncology and Palliative Medicine, Nordland Hospital, Bodø, Norway; ^2^ Department of Clinical Medicine, Faculty of Health Sciences, UiT – The Arctic University of Norway, Tromsø, Norway; ^3^ Department of Radiation Oncology, University Hospital Zurich, University of Zurich, Zurich, Switzerland; ^4^ Department of Radiation Oncology, Medical Center, Medical Faculty, University Freiburg, Freiburg, Germany

**Keywords:** brain metastases, radiotherapy, best supportive care (BSC), surgery, prognosis

## Abstract

Biologically younger, fully independent octogenarians are able to tolerate most oncological treatments. Increasing frailty results in decreasing eligibility for certain treatments, e.g., chemotherapy and surgery. Most brain metastases are not an isolated problem, but part of widespread cancer dissemination, often in combination with compromised performance status. Multidisciplinary assessment is key in this vulnerable patient population where age, frailty, comorbidity and even moderate additional deficits from brain metastases or their treatment may result in immobilization, hospitalization, need for nursing home care, termination of systemic anticancer treatment etc. Here, we provide examples of successful treatment (surgery, radiosurgery, systemic therapy) and best supportive care, and comment on the limitations of prognostic scores, which often were developed in all-comers rather than octogenarians. Despite selection bias in retrospective studies, survival after radiosurgery was more encouraging than after whole-brain radiotherapy. Prospective research with focus on octogenarians is warranted to optimize outcomes.

## Introduction

The negative impact of increasing age on prognosis has already been confirmed in the recursive partitioning analysis (RPA) of historical brain metastases trials (accrual 1979-1993), the cut-off being 65 years (1). Only 13% of patients were 70 years of age or older and the mainstay of treatment was whole-brain radiotherapy (WBRT). Since then, many countries have witnessed an increase in people older than 80 years, with heterogeneous patterns of cancer incidence, comorbidity and frailty (2, 3). However, biologically younger, fully independent octogenarians are not uncommon. Prospective clinical trials are no longer inaccessible for these patients, e.g., after geriatric assessment (4, 5). As in all age groups, oncological treatment is most commonly administered outside of clinical trials, i.e. according to standard clinical practice. Regarding brain metastases, a relatively common type of distant dissemination in patients with lung or breast cancer or malignant melanoma (6, 7), special consideration must be given to cognitive function, especially in patients with well-preserved baseline function (8). A subset of octogenarians maintains normal cognitive function despite high prevalence and incidence of cognitive decline attributed to neurodegeneration. Brain metastases treatment that prolongs survival, but compromises functional independence might not be in line with octogenarians’ goals of care. Given that sophisticated and personalized management approaches exist, while age group-specific prospective trials are lacking (9), multidisciplinary assessment of pros and cons of different options is encouraged (10–12).

## Common treatment options

Rades et al. reported a retrospective analysis of WBRT, the historical standard approach that is less commonly employed now, in 94 octogenarian patients (13). Their median survival was 2.0 months and the authors proposed a survival score featuring three prognostic groups based on Eastern Cooperative Oncology Group (ECOG) performance status (PS), number of lesions (single versus multiple), and extracranial metastases (present versus absent). Nieder et al. validated these results in an independent cohort of 50 patients (14). Median survival was 2.1 months. In their study, other factors like cancer type (better survival for breast cancer and malignant melanoma) and lack of steroid treatment were significantly associated with survival too. However, the Rades et al. score resulted in useful stratification. WBRT does not result in guaranteed symptom palliation and neither is it complication-free, as recently reviewed by our group (15). Thus, consideration should be given to two alternative options: best supportive care (BSC) (16, 17), if active brain metastases treatment is unable to extend survival beyond the median observed by Rades et al. and Nieder et al., or stereotactic radiosurgery (SRS) if the prognostic tools and the clinicians’ multidisciplinary assessment predict survival clearly beyond 2 months. Regardless of combination of prognostic features, ECOG PS 0-2 is required to become part of a subgroup with longer survival.

**Figure 1:**
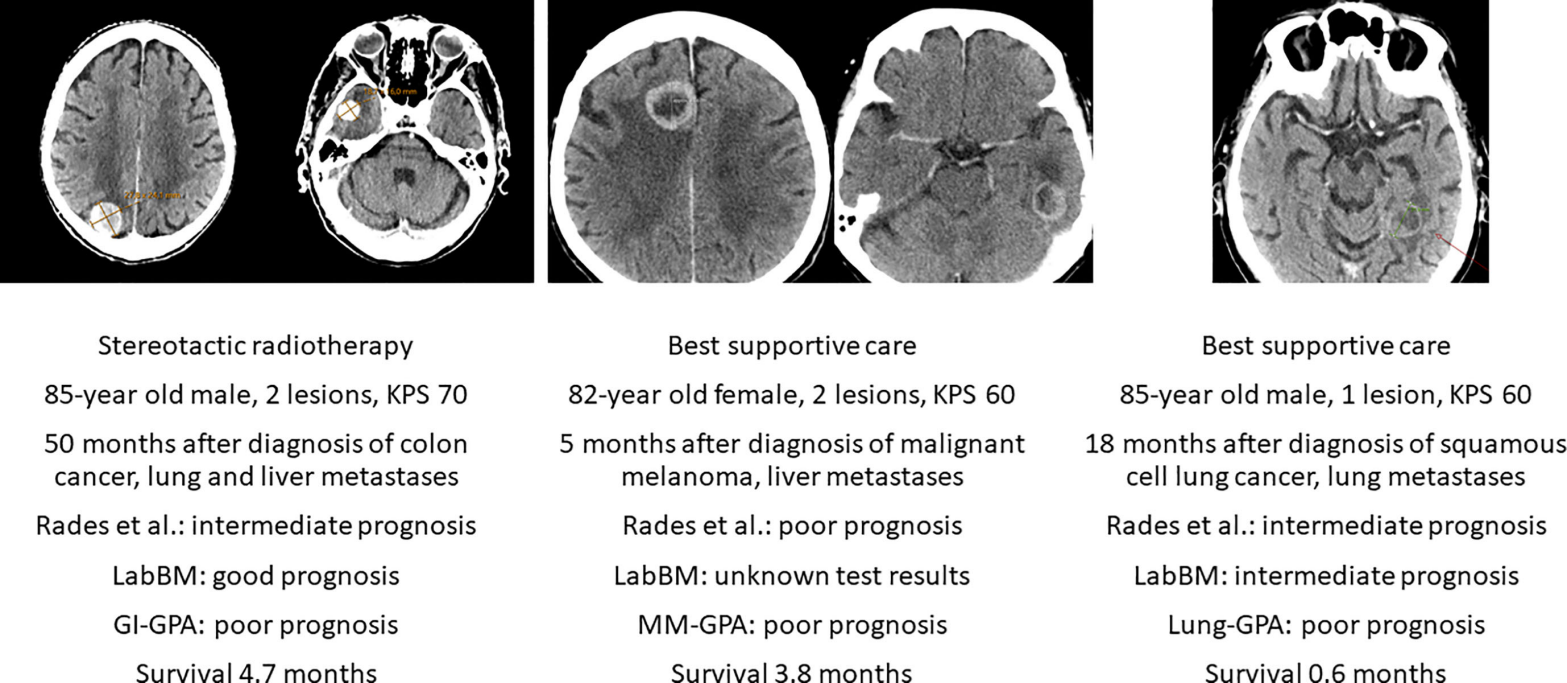
Axial computed tomography scans of three deceased patients with known survival outcome managed with stereotactic radiotherapy or best supportive care without systemic therapy after diagnosis of brain metastases. KPS, Karnofsky performance status. LabBM (23) and graded prognostic assessment (GPA) were calculated as described in the original studies (24).

Encouraging results were achieved with SRS, as suggested by a case-matched study comparing treatment results for patients 80 years of age or older versus patients 65-79 years of age (18). Overall, 165 patients were 80+ years old. Median survival time was shorter in these patients (5.3 months) than in the younger, matched group (6.9 months). However, this difference was not statistically significant (HR 1.1, 95% CI 0.9-1.4, p=0.2). A different study included 106 patients age 80 years and older who received SRS (19). The median survival was 7.1 months. Six-month and 12-month rates of local tumor control (per lesion) were 94% and 89%, respectively. Repeat SRS, salvage WBRT and surgical resection were subsequently required in 25, 4 and 1 patient, respectively. Karnofsky PS ≥ 70, controlled primary disease/no extracranial metastases and female sex were independent factors predicting better survival. Tumor volume >2 mL was the only factor predicting a higher rate of local failure. Chen et al. reported a retrospective study suggesting that WBRT was associated with increased toxicity compared with SRS in elderly and very elderly (80+) patients with brain metastases (20). Other authors have also confirmed that SRS is efficacious and safe in this population (21), albeit in absence of prospective longitudinal cognitive and quality of life analyses. The fact that additional salvage treatment might be needed after SRS is well known from the literature and not age-dependent (22). Previous limitations regarding maximum number of lesions eligible for SRS (often 3-4) are not stringently applied anymore (6, 7, 10). **Figure 1** shows case-based recommendations for the common scenarios of SRS and BSC.

## Additional treatment options

**Figure 2:**
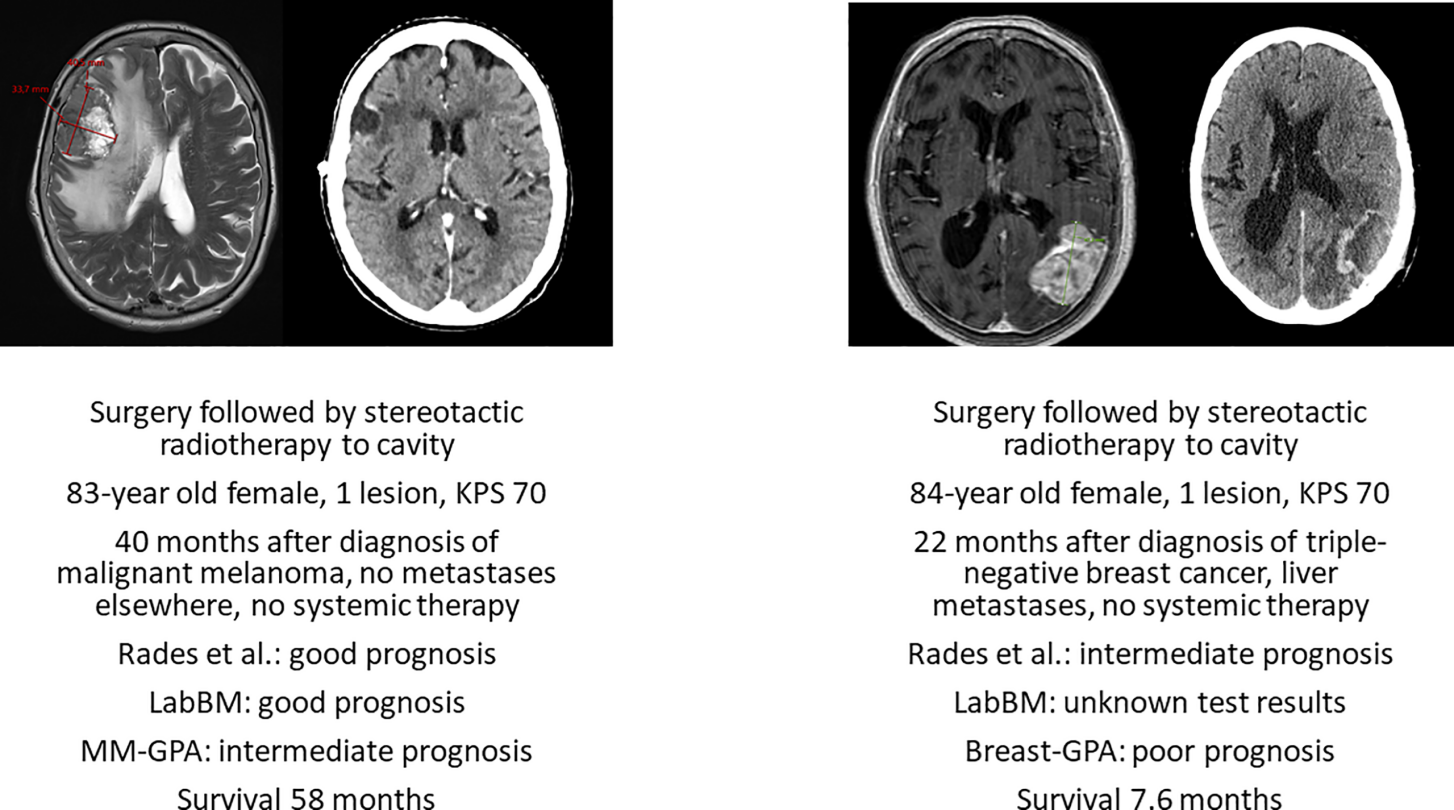
Axial pre-operative magnetic resonance imaging scans and post-operative radiation treatment planning scans of two deceased patients with known survival outcome managed with surgery and cavity-confined stereotactic radiotherapy without systemic therapy after diagnosis of brain metastases. KPS, Karnofsky performance status. LabBM (23) and graded prognostic assessment (GPA) were calculated as described in the original studies (24).

Neurosurgical resection should be considered in medically operable patients whose survival can be extended by surgery, if radiotherapy is less likely to result in equivalent outcome. The combination of a single, large and accessible brain metastasis and absent/well controlled extracranial disease might prompt the multidisciplinary team to recommend surgery, as illustrated in **Figure 2**. Surgery was evaluated in a retrospective analysis of the Nationwide Inpatient Sample (1998–2005) published in 2011 (25). Age older than 80 years and higher Charlson comorbidity scores were found to be important prognostic factors for inpatient outcome. Therefore, thorough pre-operative assessment is necessary to confirm the appropriateness and safety of this approach (26). Post-operative irradiation of the cavity/tumor bed (27, 28) can be offered also in octogenarians.

**Figure 3:**
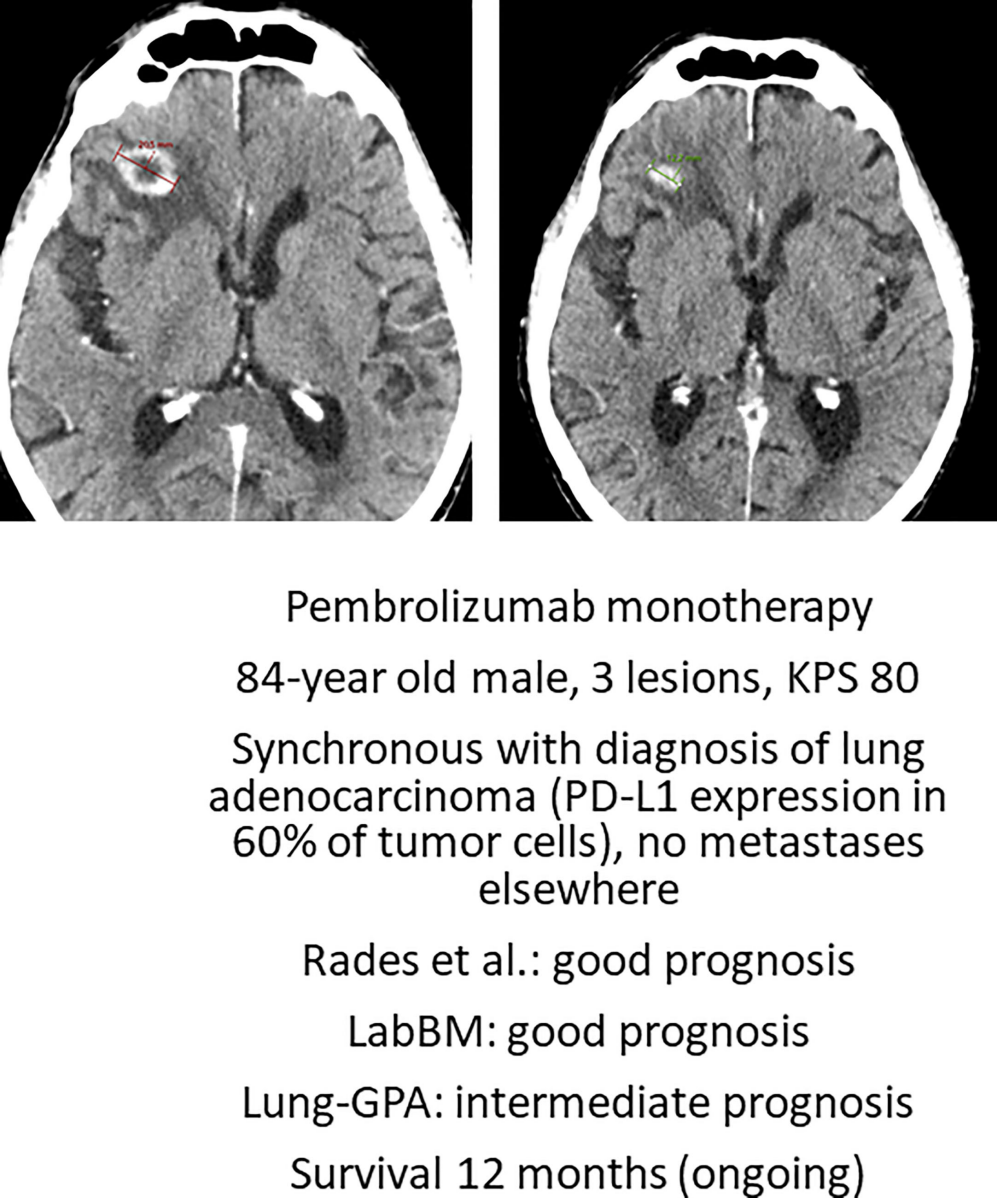
Axial computed tomography scan before Pembrolizumab and after 2.5 months of treatment. This patient is still alive. KPS, Karnofsky performance status; PD-L1, programmed death ligand 1. LabBM (23) and graded prognostic assessment (GPA) were calculated as described in the original studies (24).

**Figure 4:**
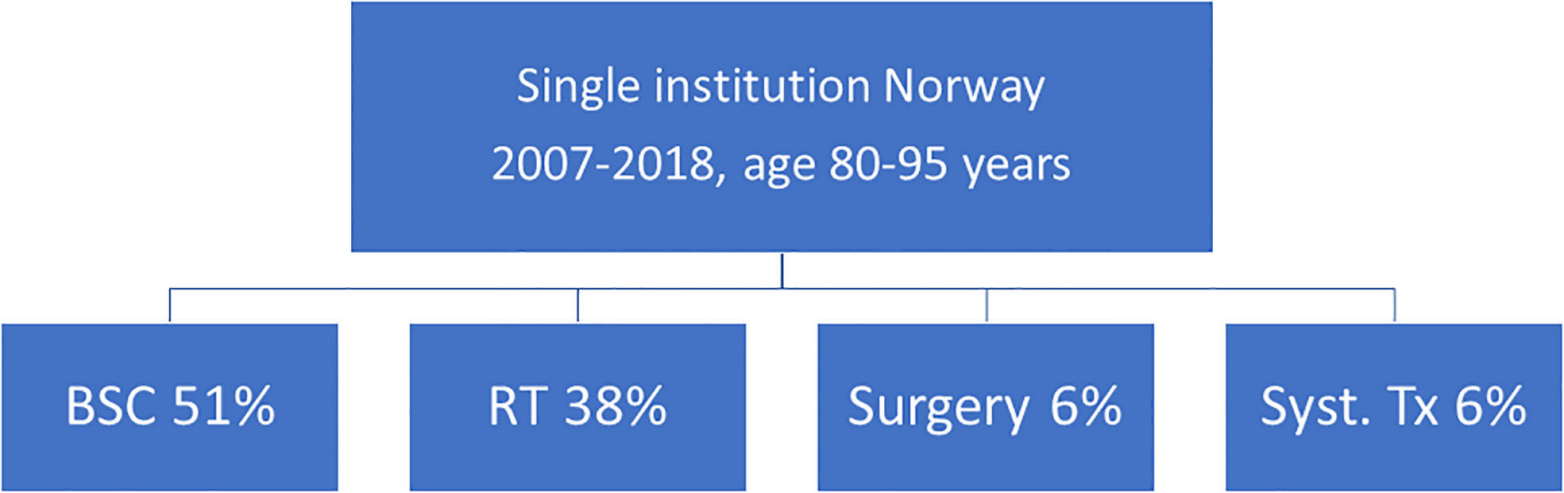
Approaches selected in one of the authors’ institutions. All patients with brain metastases were monitored in a continuously updated database (14, 17). BSC, best supportive care; RT, radiotherapy; Tx, treatment.

Deferring local treatment and tailoring it to patterns of extra- and intracranial response and availability of further lines of systemic treatment might be an option for octogenarians eligible for upfront systemic therapy (29). The phase II OCEAN study of osimertinib for radiotherapy-naive brain metastases from NSCLC (sensitizing EGFR mutation-positive) included patients with an age range of 41 to 84 years (30). The ALEX trial in patients with a different target (treatment-naive advanced anaplastic lymphoma kinase mutation-positive (ALK+) NSCLC) reported an age range of 18-81 years (31). The upper limit was identical in the phase 2 study of patients with metastatic melanoma and at least one measurable, non-irradiated brain metastasis (tumor diameter, 0.5 to 3 cm) and no neurologic symptoms who received nivolumab plus ipilimumab for up to four doses, followed by nivolumab (32). Overall, most patients in these trials were considerably younger, resulting in sparse, if any, evidence for octogenarians. Such patients were not included at all in several studies of human epidermal growth factor receptor 2-positive breast cancer and brain metastases (33–35). Even if dedicated studies for octogenarians are needed to provide firm conclusions, individual decisions for primary systemic therapy are justified (**Figure 3**), as also reflected in one of the authors’ single-institution patterns of care analysis (**Figure 4**). Six percent of these Norwegian patients were managed with primary systemic therapy.

## Conclusions

BSC was the preferred strategy in a large proportion of patients at Nordland Hospital. The longest observed survival was 6.1 months in octogenarians managed with BSC. Given that survival after WBRT was disappointing (13, 14), more efficacious, yet function-preserving SRS (or fractionated variants) should be considered, if KPS is ≥70 and active treatment is needed. Median survival in the literature was around 6 months. Selected patients with good KPS might benefit from surgical resection (large, symptomatic metastasis) or primary systemic therapy tailored to specific targets (small, asymptomatic metastases; simultaneous extracranial activity needed). As illustrated in the Figures, prognostic assessment is still imperfect and inconsistent between different scores (often developed in all-comers). Scores alone are not sufficient for decision-making, in part because frailty and comorbidity are not included in commonly used scores, despite their important impact on oncological treatment choices in the elderly and oldest old. Multidisciplinary assessment is key in such a vulnerable patient population where age, frailty, and even moderate additional deficits from brain metastases or their treatment may result in immobilization, hospitalization, need for nursing home care, termination of systemic anticancer treatment etc. Often, patient caregivers can supplement important information during decision-making and definition of the goals of treatment. If the oncologist in charge lacks confidence in a patient’s ability to tolerate treatment or provide appropriate consent, geriatric assessment should be incorporated during preparation of attempted treatment (36, 37).

## Data availability statement

The data analyzed in this study is subject to the following licenses/restrictions: Our institutional brain metastases dataset (Nordland Hospital) is available for external analyses on reasonable request from the corresponding author. Requests to access these datasets should be directed to CN, carsten.nieder@nlsh.no.

## Ethics statement

Ethical review and approval was not required for the study on human participants in accordance with the local legislation and institutional requirements. The patients/participants provided their written informed consent to participate in this study.

## Author contributions

All authors contributed to the study conception and design. Material preparation, data collection and analysis were performed by CN. The first draft of the manuscript was written by CN. and all authors commented on previous versions of the manuscript. All authors contributed to the article and approved the submitted version.
